# Dysregulated Immune Activation in Second-Line HAART HIV+ Patients Is Similar to That of Untreated Patients

**DOI:** 10.1371/journal.pone.0145261

**Published:** 2015-12-18

**Authors:** Milena S. Espíndola, Leonardo J. G. Lima, Luana S. Soares, Maira C. Cacemiro, Fabiana A. Zambuzi, Matheus de Souza Gomes, Laurence R. Amaral, Valdes R. Bollela, Olindo A. Martins-Filho, Fabiani G. Frantz

**Affiliations:** 1 Faculdade de Ciencias Farmaceuticas de Ribeirao Preto, Universidade de Sao Paulo, Ribeirao Preto, SP, Brazil; 2 Laboratorio de Bioinformatica e Analises Moleculares – INGEB / FACOM, Universidade Federal de Uberlandia, Patos de Minas, MG, Brazil; 3 Faculdade de Medicina de Ribeirao Preto, Universidade de Sao Paulo, Ribeirao Preto, SP, Brazil; 4 Laboratorio de Biomarcadores para Diagnostico e Monitoramento, Centro de Pesquisas Rene Rachou, FIOCRUZ, Belo Horizonte, MG, Brazil; University of Pittsburgh, UNITED STATES

## Abstract

**Background:**

Successful highly active antiretroviral therapy (HAART) has changed the outcome of AIDS patients worldwide because the complete suppression of viremia improves health and prolongs life expectancy of HIV-1+ patients. However, little attention has been given to the immunological profile of patients under distinct HAART regimens. This work aimed to investigate the differences in the immunological pattern of HIV-1+ patients under the first- or second-line HAART in Brazil.

**Methods:**

CD4+ T cell counts, Viral load, and plasma concentration of sCD14, sCD163, MCP-1, RANTES, IP-10, IL-1β, IL-6, TNF-α, IL-12, IFN-α, IFN-γ, IL-4, IL-5, and IL-10 were assessed for immunological characterization of the following clinical groups: Non-infected individuals (NI; n = 66), HIV-1+ untreated (HIV; n = 46), HIV-1+ treated with first-line HAART (HAART 1; n = 15); and HIV-1+ treated with second-line HAART (HAART 2; n = 15).

**Results:**

We found that the immunological biosignature pattern of HAART 1 is similar to that of NI individuals, especially in patients presenting slow progression of the disease, while patients under HAART 2 remain in a moderate inflammatory state, which is similar to that of untreated HIV patients pattern. Network correlations revealed that differences in IP-10, TNF-α, IL-6, IFN-α, and IL-10 interactions were primordial in HIV disease and treatment. Heat map and decision tree analysis identified that IP-10>TNF-α>IFN-α were the best respective HAART segregation biomarkers.

**Conclusion:**

HIV patients in different HAART regimens develop distinct immunological biosignature, introducing a novel perspective into disease outcome and potential new therapies that consider HAART patients as a heterogeneous group.

## Introduction

Systemic inflammation plays a central role in the HIV pathogenesis of untreated patients and correlates with disease progression and morbidity [1,2]. Clinically, CD4+ T cells counts and the viral loads are together the main parameters of status for disease evaluation [3,4]. The inclusion of highly active antiretroviral therapy (HAART) has historically changed the perspective of HIV infection worldwide. The complete suppression of viral load and partial restoration of CD4+ T cells improve health and prolongs the life expectancy of infected patients [5,6], turning AIDS into a chronic disease. Additionally, a large number of new plasma biomarkers, such as the soluble monocyte and macrophage activation biomarkers sCD14 and sCD163, inflammatory cytokines, chemokines, and coagulation factors are important indicators of immune activation. These factors correlate with disease susceptibility and progression, and morbidity and mortality in treated and untreated patients [7–12].

HIV treatment is life-long and requires an optimal adherence in order to delay the rise of drug resistance. Most patients are under the first-line therapy that combines two nucleoside reverse transcriptase inhibitors (NRTIs) and one non-nucleoside reverse transcriptase inhibitor (NNRTI). Early in treatment positive clinical and immunological outcomes are observed while viral load drops significantly [13–17]. However, in situations when the use of the NNRTIs is not recommended or in virologic suppression failure after first-line regimen, the protocol is to proceed to the second-line regimen, which indicates the replacement of the NNRTI with a Protease Inhibitor (PI) [18].

Nevertheless, a number of studies regarding the immunological profile of HIV+ patients do not consider treatment regimens while HAART patients are seen as a closed and unified group [19–21]. Given the fact that little attention has been given to investigating the immunological profiles of AIDS patients under different regimes, this work aims to identify differences in the immunological profile of patients and potential association with disease outcome in HIV+ patients under the first- and second-line HAART regimens. By using systems biology, we show that the immunological biosignature of HIV patients changes according to the HAART regimen, which can provide insights on the patient’s clinical status.

## Materials and Methods

### Ethical Aspects

The study was approved by the Ethics Committee from the Hospital das Clínicas de Ribeirão Preto and FMRP-USP (Protocols #11399/2012; #9817/2012; #9818/2012) and from CEP/FCFRP-USP (Protocol #276/2012). All patients signed an informed written consent form in accordance with the guidelines established by the Brazilian National Health Council.

### Study Population

Untreated HIV-1-infected patients (n = 46), HIV+ patients under first-line treatment (HAART 1; n = 15), and HIV+ patients under second-line treatment (HAART 2; n = 15) from both sexes and aged between 18 and 65 years, were recruited from Hospital das Clínicas de Ribeirão Preto, FMRP-USP, São Paulo, Brazil. The two groups of treated patients were included considering that viremia was undetectable. The most frequent clinical criterion used for indicating the second-line regimen in our group of patients was side effects developed to the first-line regimen, or pre-existing conditions that precluded use of Efavirenz, such as history of previous mental health disorders. Non-infected individuals (NI) (n = 66) were volunteer healthy blood donors of the Hemotherapy Center of Ribeirão Preto in São Paulo, Brazil, aged between 18 and 65 years, HIV-1 negative and without history of chronic illness or drug use. Treated patients’ regimen details, basic characteristics of HIV-1-infected individuals and healthy donors, and detailed information about HAART treated groups, including duration of infection, number of years under treatment, and nadir CD4+ T cell counts are listed in S1, S2 and S3 Tables, respectively.

### Biomarker Measurement

Blood specimens were collected using Heparin blood collection tubes (BD Biosciences, San Diego, CA). Plasma separation was performed strictly according to the manufacturer’s protocol. A customized multiplex kit was used to measure MCP-1, RANTES, IP-10, IL-1β, IL-6, TNF-α, IL-12, IFN-α, IFN-γ, IL-4, IL-5, and IL-10 (12-plex, EMD Millipore Corporation, Billerica, Massachusetts, USA). A fluorescent bead-based instrument (Luminex^®^ MAGPIX^®^ System; Luminex Corporation, Austin, Texas, USA) was used to read each multiplex plate. Luminex bead-based data were analyzed using Milliplex Analyst software v3.5 (Millipore; VigeneTech Inc., Boston, Massachusetts, USA) and a three-parameter logistic curve fit. Soluble CD14 and sCD163 concentration were measured in plasma using the DuoSet ELISA kit (R&D Systems, Minneapolis, Minn), according to the manufacturer’s instructions.

CD4+ T-cell counts for HIV-1+ patients were measured with an FACS Calibur flow cytometer (Becton-Dickinson, USA). HIV-1 RNA levels in plasma (viral loads) were determined by Real Time PCR for HIV-1 (Abbott Molecular).

### Data Analysis

This was a descriptive transversal study that applied five data analysis approaches for observational investigation of the immunological profile associated with distinct HIV-1+ treatment regimens: (1) conventional statistical analysis, (2) biomarker signature analysis, (3) biomarker network, (4) heat map group segregation analysis, and (5) decision tree analysis. These approaches have been shown as relevant to detect, with high sensitivity, putative minor changes in the immunological profiles that are not detectable by conventional statistical approaches [22–25].

### Conventional Statistical Analysis

Differences between groups were first evaluated to test normality. The Mann-Whitney test was used to compare data between NI and HIV+ patients. Additional analyses among HIV subgroups were performed by the Kruskal-Wallis test, followed by Dunns’ multiple comparison test. GraphPad Prism 5.0 software (Graph-Pad Software, San Diego, CA, USA) was used for calculation of statistical analyses and differences were considered significant at p < 0.05.

### Biomarker Signature Analysis

The cytokine profile was first assessed to identify the cytokine production pattern of each individual, as previously shown [23,24]. The whole universe of data of each biomarker was initially used to calculate the global median value used as the cut-off to classify patients as a “low” or “high” producer of a given biomarker. The cut-off values are detailed in S1 Fig. Once the cut-off for each biomarker was established, we selected the high producers of each biomarker and assembled the data using black-and-white scale diagrams to calculate the frequency of those for each group. When considering disease progression status, the cut-off values for progression biomarkers viral load, CD4+ T cell count, sCD163, and sCD14 were calculated as the global median value from untreated HIV-1+ group, and patients were classified as “Putative Slow Progressors” or “Putative Rapid Progressors” as detailed in S2 Fig. Significant data (> 50%) were then highlighted in bold/underline format. Radar charts characterize the overall frequency of patients with high levels of a given immunological biomarker.

### Biomarker Network

Spearman rank correlation test was performed to assess the association between levels of immunological biomarkers in each group. In all cases, significance was considered at p<0.05. All tests were provided by GraphPad Prism version 5.0 (San Diego, CA). Significant correlations representing the interaction between the biomarkers tested were compiled using the open source software Cytoscape (version 2.8; http://www.cytoscape.org), as previously reported [26]. Biomarker networks were constructed using circle layout. Connecting edges display the underscore of correlation index (*r*) as negative (*r* < 0), weak (*r* ≤0.35), moderate (0.36 ≥ *r* ≤ 0.67), and strong (*r* ≥ 0.68), as proposed previously [27].

### Heat Map and Decision Tree

The heat map analysis was carried out using the heatmap.2 function in the R (Project for Statistical Computing Version 3.0.1) and gplots package with the default clustering parameters. All analyses were performed using customized functions available from Bioconductor packages. After dataset analysis, a decision tree was generated for each heat map. The C4.5 algorithm [28] was used to build the decision tree from WEKA implementation software (Waikato Environment for Knowledge Analysis, version 3.6.11, University of Waikato, New Zealand), using default J48 parameters [29]. The decision trees, the most widely used machine learning algorithms, were used to select the minimal set of phenotypic features that efficiently segregated the groups. This method analyzes all the phenotypic attributes in the training set and selects the most relevant attribute that maximizes the information gain as the root node. The method continues searching for additional attributes for group segregation. In order to estimate the classification accuracy of the decision tree models on new data with unknown class labels, we used a 10-fold cross validation methodology available in the WEKA software.

## Results

### Untreated HIV patients have systemic increments of immune biomarker production

**Fig 1:**
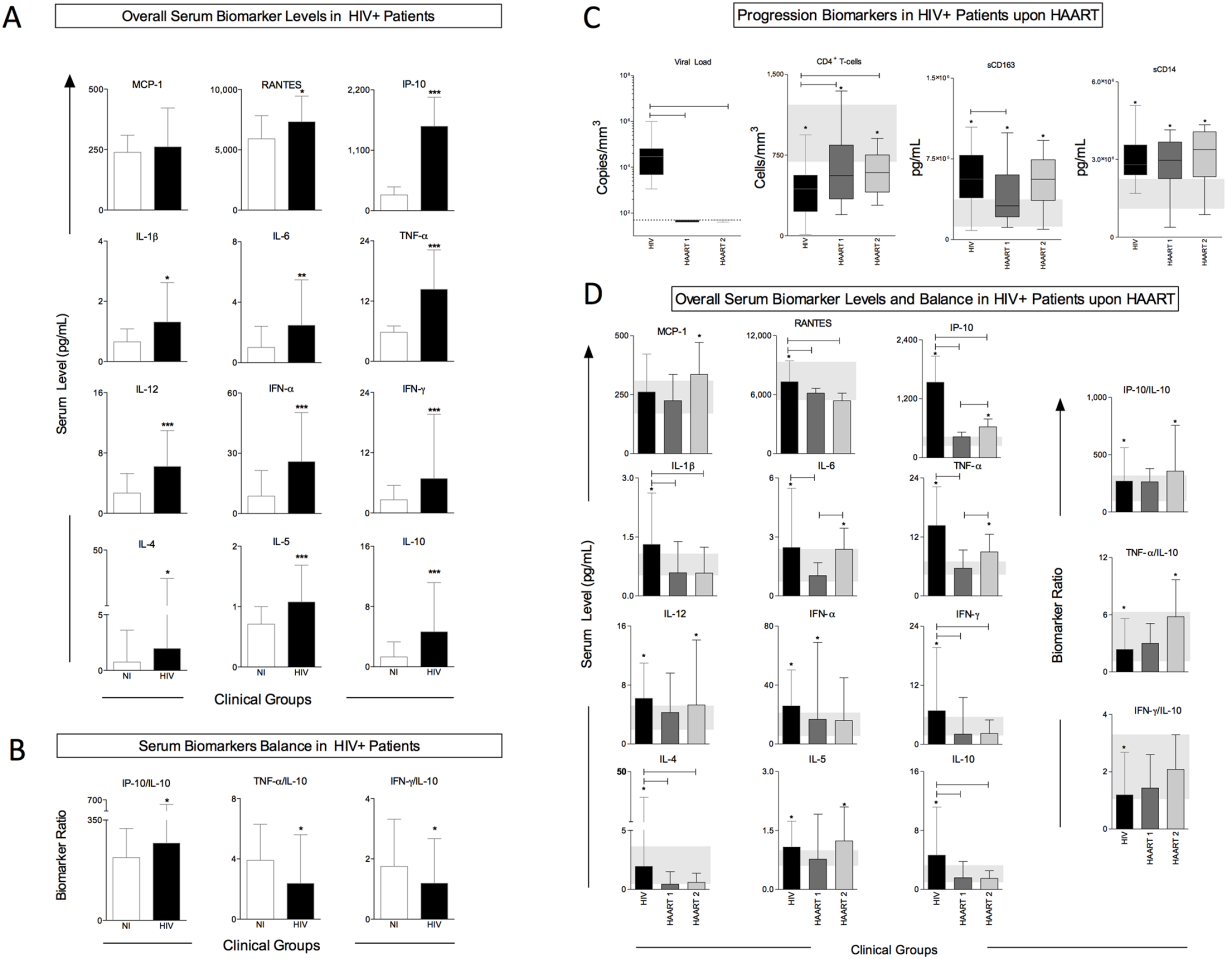
Immunological and progression biomarkers overall profile of untreated and treated HIV+ patients under different antiretroviral regimens. A) Overall immunological profile of untreated HIV+ patients; B) Biomarker ratio of untreated HIV+ patients; C) Progression biomarkers in HIV+ patients under HAART; D) Overall immunological profile of HIV+ patients under HAART. Plasma progression biomarkers and immunological biomarkers were obtained by quantification of viremia, CD4+ T cells counts, sCD14, sCD163, and MCP-1, RANTES, IP-10, IL-1β, IL-6, TNF-α, IL-12, IFN-α, IFN-γ, IL-4, IL-5, and IL-10 in plasma. Results are expressed as median ± interquartile range. ***p<0.001; **p<0.01; *p<0.05 vs. NI; p<0.05 between indicated groups. The interquartile range of NI group is expressed as a gray rectangle under the bars. NI: Non-infected individuals. HIV: Untreated HIV+ patients.

The level of 12 plasma cytokines and chemokines was assessed in order to characterize the overall immune profile of untreated HIV+ patients. Our results show increased plasma concentration of all immune biomarkers evaluated, except for MCP-1, in the plasma of untreated HIV+ patients compared to non-infected individuals (NI), indicating a distinctive immune activation state in untreated HIV+ patients (Fig 1A). Among the biomarkers tested, levels of inflammatory, adaptive, and anti-inflammatory cytokines were all higher. We also observed that IP-10/IL-10 ratios were increased in HIV+ patients while TNF-α/IL-10 and IFN-γ/IL-10 were reduced (Fig 1B).

### HIV+ patients under different HAART regimens display similar levels of progression biomarkers whereas immunological plasma varies

We used the classical parameters CD4+ T-cell counts and viral load to classify disease progression in untreated patients. We also evaluated serum levels of sCD14 and sCD163 in untreated and treated patients under either first-line or second-line treatments.

Undetectable levels of viral load were observed in plasma from patients under either HAART regimen, confirming the efficacy of viral load suppression in our tested cohort. Untreated and treated HIV+ patients had reduced CD4+ T cells counts compared to healthy individuals; however, patients under either HAART regimen showed a significant recovery of CD4+ T cells. The levels of progression biomarkers sCD163 and sCD14 were higher in all HIV+ patients, although patients on HAART 1 had reduced serum levels of sCD163 when compared to untreated patients (Fig 1C).

The use of antiretroviral therapy significantly reduced serum levels of immunological biomarkers RANTES, IP-10, IL-1β, IFN-γ, IL-4, and IL-10. Serum levels of IL-6 and TNF-α were reduced only in patients under HAART 1 regimen, if compared to untreated HIV+ patients. Between the two treatment regimens, significant differences were observed in the levels of IP-10, IL-6, and TNF-α, which are key immunological markers associated with the progression and susceptibility to HIV infection. We found that individuals under HAART 2 had significantly higher serum concentration of the three cytokines, if compared to individuals under HAART 1. In addition, the ratios of IP-10/IL-10 and TNF-α/IL-10 from untreated HIV and HAART 2 patients were higher than the levels observed in non-infected individuals, while no differences were observed in the ratios from patients under HAART 1. Our results also show that the levels of immunological biomarkers in the HAART 1 group were similar to those of NI individuals, except for the production of IFN-α (Fig 1D).

### HAART 2 patients showed a stronger inflammatory biosignature than HAART 1 patients

To better understand and characterize the relationship between different HIV clinical groups and their respective immune biosignature, we used categorical analysis strategies.

**Fig 2:**
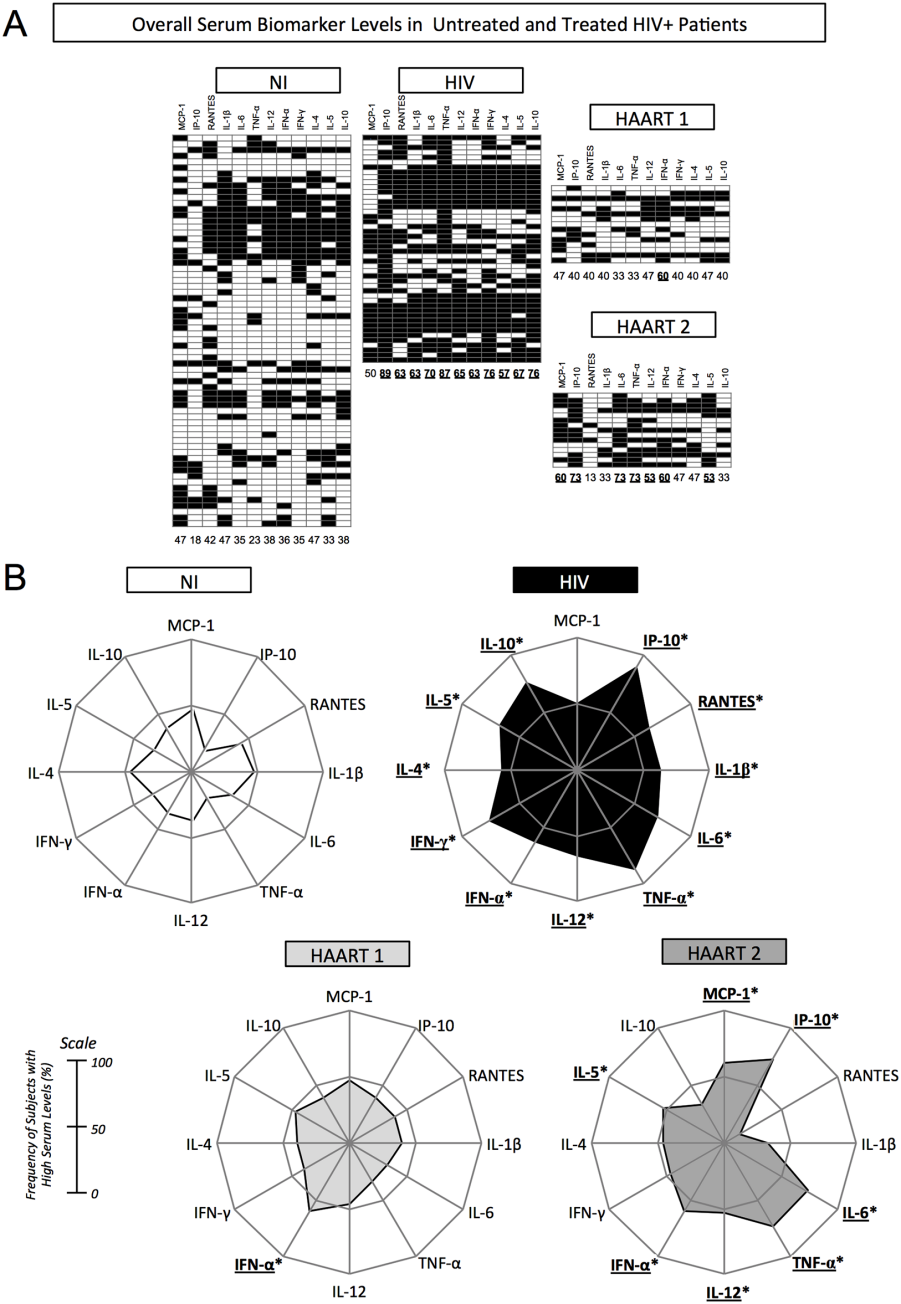
Immunological biosignature of NI, untreated, and treated HIV+ patients (HAART 1 and HAART 2). Plasma concentration of MCP-1, RANTES, IP-10, IL-1β, IL-6, TNF-α, IL-12, IFN-α, IFN-γ, IL-4, IL-5, and IL-10 was used for categorical classification of NI, HIV, HAART 1, and HAART 2 groups between high and low producers of a given immunological biomarker. A) Black and white diagrams represent high and low producers of each cytokine, respectively. Each lane represents a cytokine and each block represents the pattern of cytokine production of each patient. Numbers below each lane represent the frequency of high producers of the cytokine tested. B) Radar charts summarize the percentage of high producers of each clinical group studied. When the frequency of high producers was greater than 50% (on a scale of 0–100%), the result was highlighted.

The number of subjects with high cytokine levels was compiled in a black-and-white scale diagram to determine the frequency of high producers, in each clinical group (Fig 2A). Our data show that while the NI group was mostly formed by low producers, untreated HIV+ patients exhibited an opposite profile, characterized by a higher frequency of high producers of most cytokines. On the other hand, the profile of patients under treatment was balanced, but those who received HAART 1 were mostly low producers, while patients under HAART 2 were mostly high producers of the biomarkers studied here (Fig 2A).

By using radar charts as a comparison strategy among clinical groups, we observed significant differences in the overall biomarker profile of high producers. We considered a biomarker relevant when the frequency of high producers was greater than 50% [23,24]. Therefore, we noted that NI subjects had less than 50% frequency of high cytokine producers for all the biomarkers studied here. In contrast, HIV infection was associated with a higher frequency for all biomarkers except MCP-1. HAART 1 patients showed a decrease in the overall profile of cytokine production, in which only IFN-α remained highly produced by more than 50% of patients. Furthermore, patients under HAART 2 displayed higher levels of IP-10, IL-6, TNF-α, IL-12, IFN-α, and IL-5 (Fig 2B). Our data indicate that the immune profile of patients receiving HAART 1 resembles the profile of NI individuals, while HAART 2 displays a profile closer to the one presented by untreated HIV patients.

### Segregation according to disease progression reveals that HAART 2 patients are unable to restore immune biosignature to NI pattern

Considering the distinct immune profiles of patients under different treatment regimens, we aimed to evaluate the correlation between immunological biosignature and disease progression in the studied groups. For that, we used Viral Load and CD4+ T cell counts, in addition to sCD163 and sCD14 levels as markers for disease progression (S2 Fig) and calculated the agreement percentage between each pair (S3 Fig).

**Fig 3:**
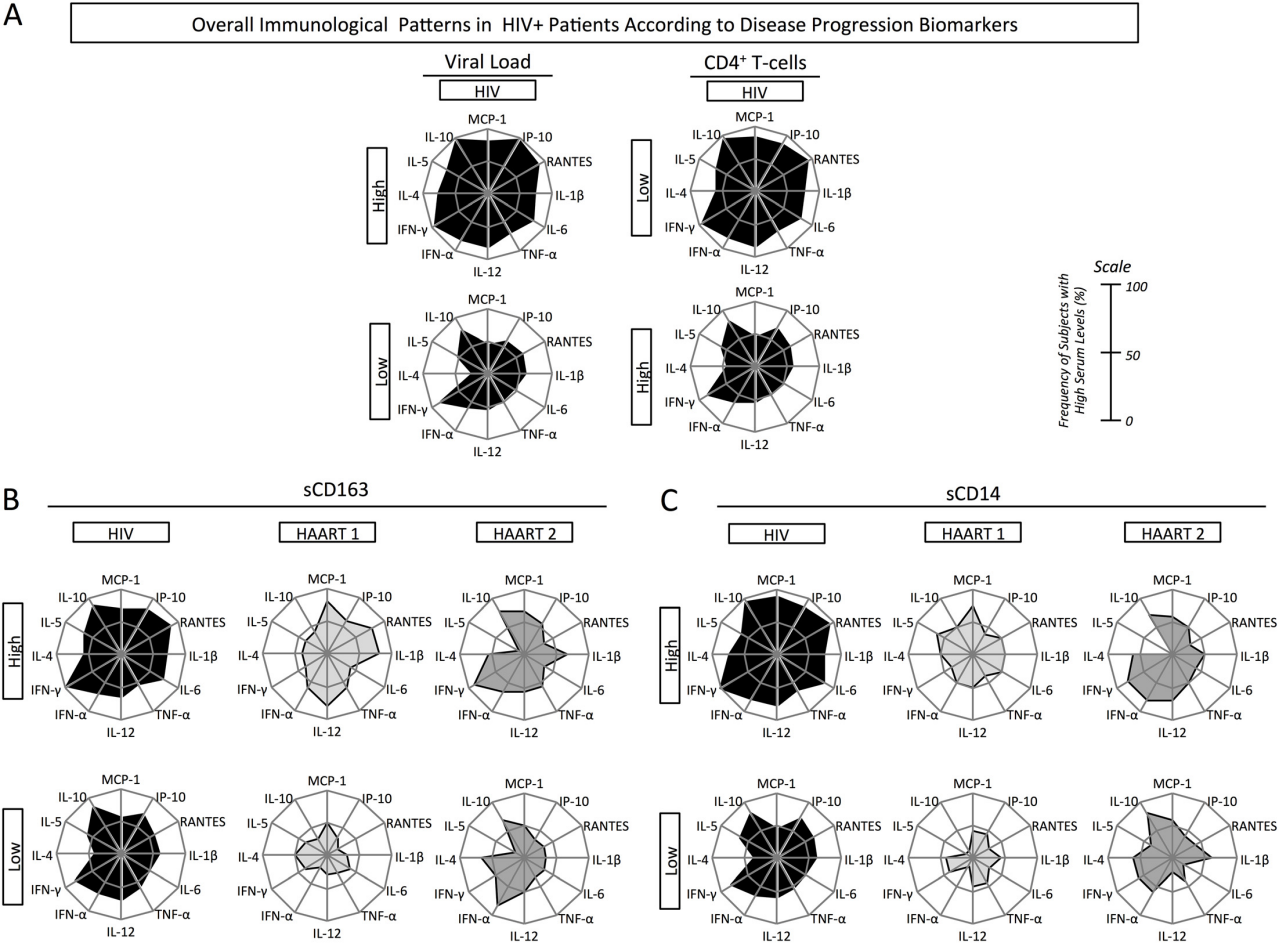
Immunological biosignature of untreated and treated HIV+ patients under two antiretroviral regimens, according to disease progression. Viral load, CD4+ T cell counts, sCD163, and sCD14 levels were used as progression biomarkers to classify HIV+ patients in patients presenting rapid (upper charts) or slow (lower charts) disease progression. Plasma concentration of MCP-1, RANTES, IP-10, IL-1β, IL-6, TNF-α, IL-12, IFN-α, IFN-γ, IL-4, IL-5, and IL-10 was used to characterize the immunological biosignature of HIV+ patients. Radar charts summarize the percentage of high producers of each group studied. A) Biosignature of untreated HIV patients according to viral load and CD4 count categorization. B) Biosignature of untreated HIV, HAART 1, and HAART 2 patients according to the levels of plasma sCD163. C) Biosignature of untreated HIV, HAART 1, and HAART 2 patients according to the levels of plasma sCD14. Significant changes in biomarker production were considered when the frequency of high producers was greater than 50% (on a scale of 0–100%).

Fig 3A shows untreated HIV profile according to high producers of biomarkers, categorized by Viral Load and CD4+ T cell count parameters. We observed that patients presenting rapid disease progression displayed an exacerbated inflammation, and the group is characterized by a higher frequency of high producers, over 75% in most cases, and by a high number of altered biomarkers. On the other hand, patients presenting slow disease progression had a moderate inflammatory status in which eight of the twelve cytokines have a frequency of high producers above 50%, but with lower intensity if compared to that observed in patients with rapid progression.

Since CD4+ T cell counts and viral load are affected by antiretroviral treatment, we used sCD163 and sCD14 levels for categorizing disease progression on treated groups. Using sCD163 as a parameter, patients with rapid disease progression under either treatment displayed moderate inflammation, and the frequency of high producers was above 50% in seven and nine biomarkers in the HAART 1 and HAART 2 clinical groups, respectively. On the other hand, strong differences were observed between treatments in individuals showing slow disease progression. HAART 1 patients showed similar profile to that of NI individuals, where none of the biomarkers were highly produced by more than 50% of patients, while all patients under HAART 2 remained in a state of moderate inflammation (Fig 3B).

By using sCD14 to classify patients according to disease progression, HAART 2 patients presenting rapid disease progression displayed moderate inflammation, in which six of twelve cytokines were highly produced by more than 50% of the patients, while among HAART 1 patients only two cytokines were highly produced by more than 50% of the patients. Likewise, HAART 1 patients presenting slow disease progression did not present any inflammatory profile, differently than HAART 2 patients who presented an overall profile of moderate inflammation, as more than 50% of the patients were high producers of six of the twelve cytokines tested (Fig 3C).

Thus, we found that antiretroviral therapy reduces inflammatory exacerbation even in patients presenting rapid disease progression. Nevertheless, immunological biosignature from HAART 1 displayed a similar pattern to NI especially among patients with slow disease progression. Conversely, HAART 2 biosignature showed moderate inflammation in patients presenting rapid disease progression that otherwise persisted in patients presenting slow disease progression, which indicates similarities between the patterns presented by patients under HAART2 and untreated patients.

### HAART regimen affects biomarker network organization of HIV+ patients

**Fig 4:**
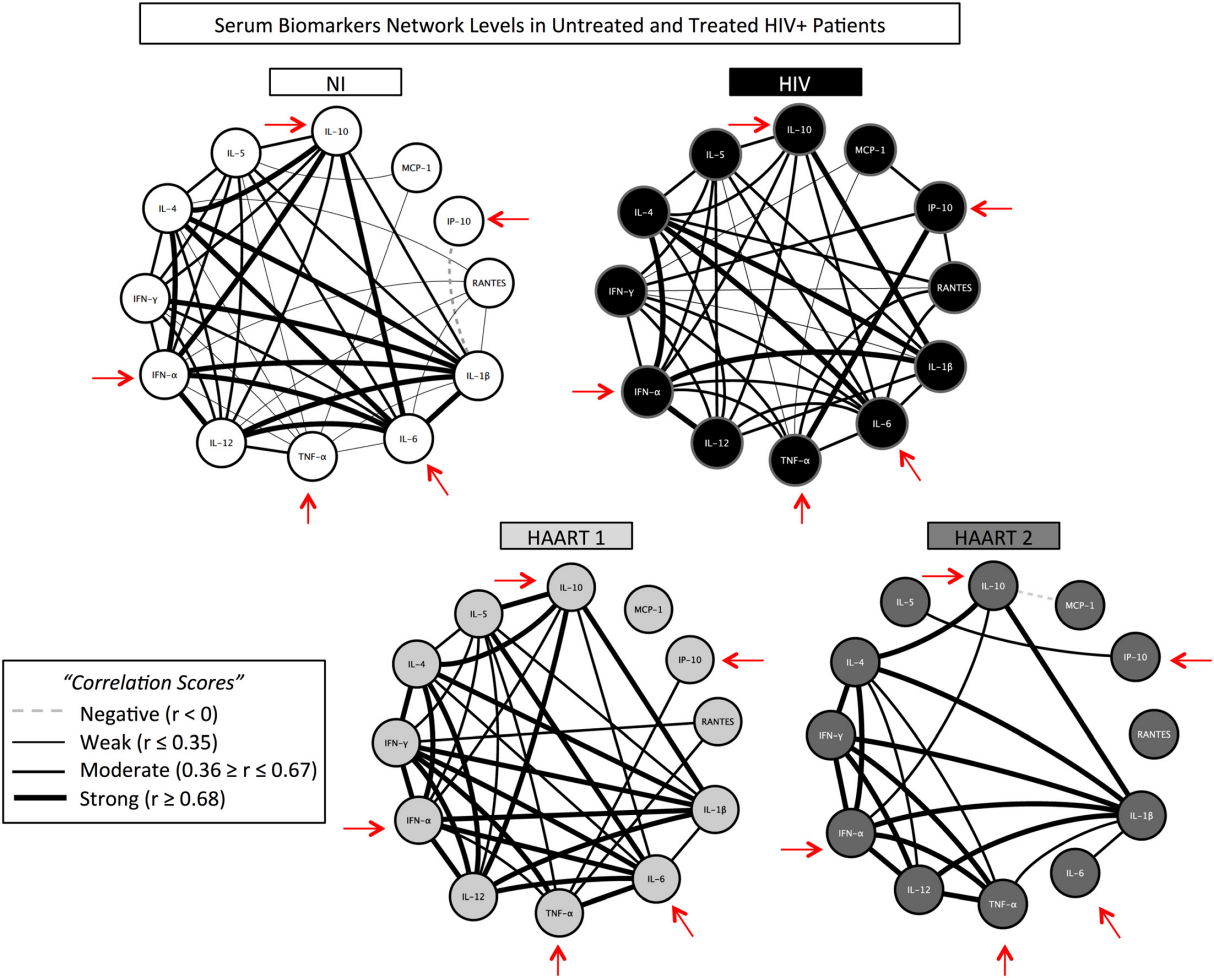
Systemic interaction of immunological biomarkers is modified in the course of HIV disease and according to treatment regimen. The network analysis shows significant correlations (p<0.05) among all the variables, which were measured after calculation of Spearman correlation for each pair of biomarkers, and are represented by lines for NI, HIV, HAART 1, and HAART 2 groups. The strength of the correlation was given by “r” value and is illustrated as negative (r < 0), weak (r ≤ 0.35); moderate (0.36 ≤ r ≤ 0.67); or strong (r ≥ 0.68). Arrows indicate the main biomarkers modified during HIV infection.

Next, we evaluated for a potential correlation among immunological biomarkers (Fig 4). We observed that NI individuals presented a network with many connections, in which the cytokines IL-1β, IL-6, IFN-α, IL-4, and IL-10 correlated with each other through strong interactions. We also observed strong and moderate correlations involving IL-12, weak correlations involving TNF-α, and one negative correlation associated with IP-10. Compared to the NI network, HIV infection induces changes in immune connections, observed through weaker connections involving IL-6, IFN-α, and IL-10; and through stronger connections involving TNF-α and IP-10. On the other hand, in the networks of HIV+ patients under HAART 1, IL-12 and IFN-γ formed stronger connections and MCP-1 showed no connection with other cytokines. Notably, we observed the recovery of patterns modified during infection, such as the IP-10, IL-6, and IL-10 to a pattern similar to that of NI, in patients under HAART 1. Oppositely, HAART 2 treatment was associated with a significant loss of connection, generating a distinctive immune network. Interactions involving IL-6, IL-10, MCP-1, and RANTES are among the main biomarkers that lost important connections, while connections involving IL-1β and IFN-α were preserved, when compared to NI individuals (Fig 4). Therefore, this approach allowed us to identify important differences involving IP-10, TNF-α, IL-6, IFN-α, and IL-10 interactions that play important roles in AIDS and are a hallmark between the two treatments here analyzed.

### Heat map analysis shows immunological biomarker segregation of HAART clinical groups

Once the main biomarkers were identified through analysis of network connections, we classified them by their association with each clinical group.

**Fig 5:**
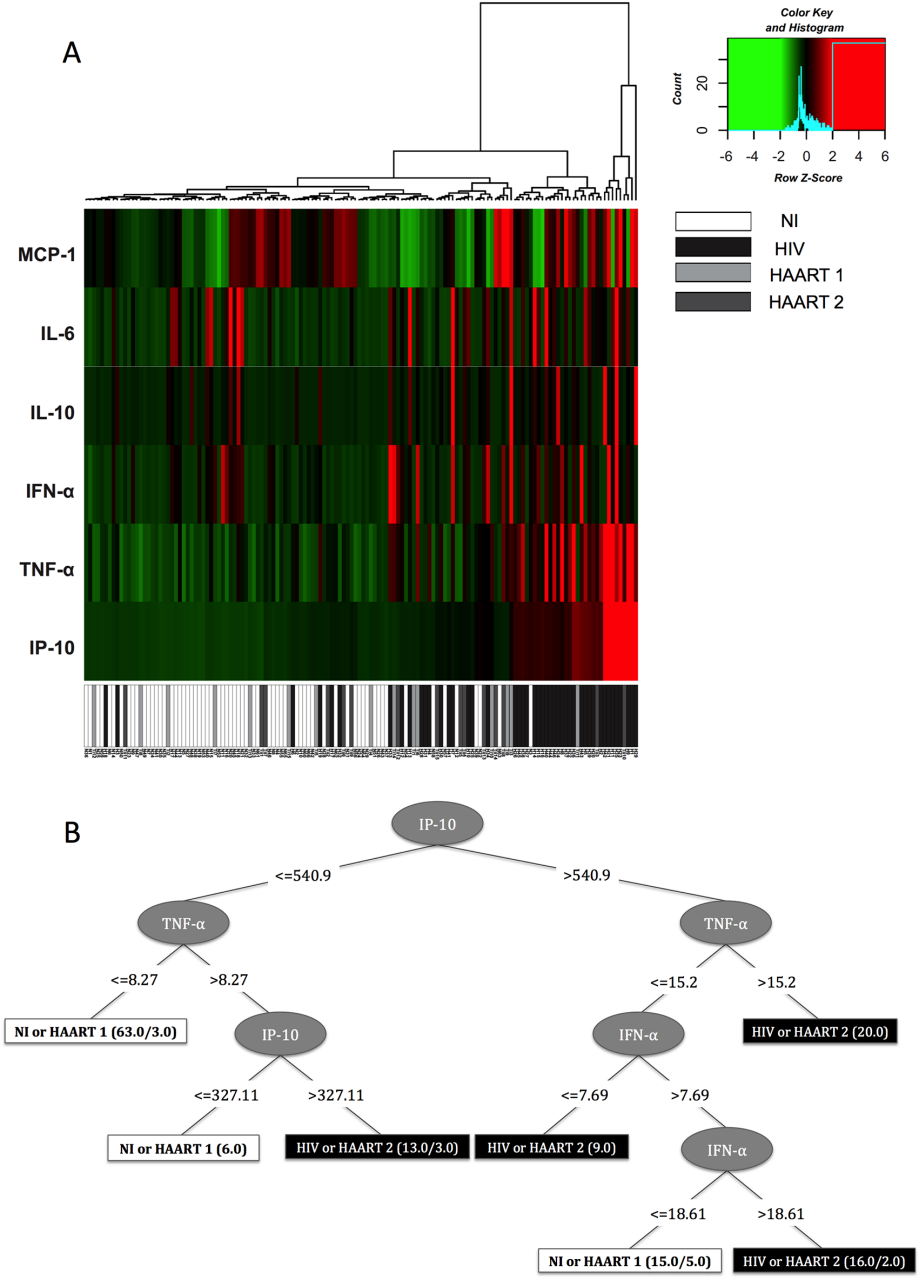
The expression pattern of the main immunological biomarkers defines a segregation profile upon infection and between different treated HIV groups. A) The values of IP-10, IL-6, TNF-α, IFN-α, IL-10, and MCP-1 were used to calculate the Z-score for each cytokine produced by subjects individually, for further generation of a heat map. Row Z-score scaled from -6 to +6 and is illustrated as green, black, and red colors. B) The values of IP-10, IL-6, TNF-α, IFN-α, IL-10, and MCP-1 were used for the decision tree dataset construction. The decision tree was generated considering the clustering of NI and HAART 1 as one group, and HAART2 and HIV as another. The numbers beside the group names indicate the correct/incorrect ranking of the records.

The expression profile of biomarkers IP-10, IL-6, TNF-α, IFN-α, IL-10, and MCP-1, was determined in each patient and plotted in a heat map as shown in Fig 5A.

Based on this analysis, we noted that the biomarker that best distinguished between uninfected individuals and HIV+ patients, regardless of treatment, was IP-10, followed by TNF-α and IFN-α. Furthermore, the pattern of HIV patients under HAART 1 was scattered through that of NI individuals whereas patients under HAART 2 were included among untreated HIV+ patients. These results support that a distinctive immunological profile is generated in patients under different treatments, which may have great impact on the health and life expectancy of HIV+ patients.

### Decision tree analysis identifies IP-10, TNF-α, and IFN-α as the main segregation immunological biomarkers between treatments

Using the biomarkers selected previously, we constructed a decision tree in order to point out which of the biomarkers would be crucial in discriminating the groups. NI and HAART 1 individuals show a similar pattern and thus were clustered in one large group whereas untreated patients and HAART 2 patients were together in another group.

Using all 142 entries to build the decision tree, the generated structure had an average accuracy of 90.84%, ranking 129 entries correctly and missing only 13. The most important attributes for the decision tree were "IP-10" followed by "TNF-α". Notably, only these two biomarkers ranked 83 of 142 (58.45%) database entries, with a classification rate of 96.38%, ranking 63 as "NI or HAART 1", missing 3; and classifying 20 as "HIV or HAART 2 ", not missing any entries (Fig 5B).

## Discussion

This study aimed to identify differences in the immunological profile and potential association with disease outcome in HIV+ patients under the first- and second-line HAART regimens.

Immune activation in the course of HIV infection initiates during acute infection, which is characterized by a rapid spread of the virus throughout the gut-associated lymphoid tissue, damaging mucosal integrity and leading to a continuous loss of HIV-susceptible, CCR5-expressing CD4+ T cells [30–32]. In our work, we clearly detected this uncontrolled immune activation, indicated by an increase in cytokine production in untreated patients in both the adaptive and innate systems (Fig 1A). Even IL-10, a regulatory cytokine, was elevated in our untreated HIV+ patients, probably as an attempt to counterbalance the excess of proinflammatory response. The cytokines evaluated by us are highly produced in acute HIV infection and some of them, such as INF-γ, RANTES, IP-10, and IL-12, were highlighted previously by other authors as early biomarkers of immune activation that could be used to help predict viral set point variation and disease progression [9].

The access to HAART made possible a durable and perhaps life-long viral suppression, but immune reconstitution is often only partially recovered [33–35]. Once under the right treatment, patients achieve almost complete inhibition of HIV replication, immune function is improved over time, and there is near elimination of any risk for developing complications that define AIDS. Instead, the risk for developing several non-AIDS disorders is increased in HIV-infected individuals, particularly if HAART is started late [36–38]. A growing number of studies have implicated monocyte- and macrophage-related inflammatory biomarkers, sCD163 and sCD14, that are also markers of microbial translocation, as predictors and presumable causes of disease progression [7,12,39,40]. However, we did not find significant differences in the level of sCD163 and sCD14 between the two treatments, thus treated patients were considered under similar disease progression status.

Our analysis cannot rule out the fact that some other events occurring before treatment started also play a role in the immunological profile of the patient. Nevertheless, the fact that the progression biomarkers are similar among treated groups (Fig 1C) led us to assume that the regimen has a direct impact on the patient’s immunological status. Also, considering that there are other important variables that can interfere with the immune profile observed in the different treated groups, such as the level of nadir CD4+ T cell counts (that is, the lowest count before treatment), duration of infection, duration of treatment, immune recovery after treatment, and residual viremia, we performed supplemental analysis to rule out that some of these factors would play a primary role in affecting our results. Thus, we observed that the two treated groups analyzed here are composed by patients who are long-term infected (HAART 1 mean = 11.67 (8–17) years vs. HAART 2 mean = 10.13 (4–17) years), and likewise display similar duration of treatment and similar nadir CD4+ T cell counts (S3 Table). Moreover, we observed a similar pattern of CD4+ T cell reconstitution, as shown by the distribution of elevated and poor recovery that is observed in both treated groups (S4 Fig). We also performed Spearman correlation analysis, testing each possible interfering variable versus immunological plasma biomarker values and found that none of them showed a significant correlation (S4 Table), except for RANTES versus CD4+ T cell counts and RANTES versus duration of treatment, both correlations found in the HAART 2 group. Therefore, considering the overall analyses we concluded that for our group of patients none of these factors are the primary factors responsible for immune biosignature profile developed by each group. Our data support the hypothesis that the different treatments are the main events associated to different immune biosignature in each group of treated patients.

Regarding residual viremia, all treated patients included in the study had undetectable levels of viremia, which are below 50 copies/mL. Thus, although very unlikely, it cannot be rule out that residual virus replication plays a distinct role in establishing the different immune profiles observed under different treatments.

The immune biosignature of patients under HAART 1 and HAART 2 revealed the occurrence of two patterns. One pattern, composed by two groups, the NI and HAART 1, presents the same biosignature profile (Fig 2B). Together, untreated HIV patients and HAART 2 compose another pattern with similar biosignature profile. Even when we set apart patients with slow and rapid disease progression based on sCD163 and sCD14 levels (Fig 3B and 3C), the differences between these two patterns were still very clear.

Previous studies have shown that protease-inhibitor-based therapies for HIV patients might induce extravirologic modulation of immune pathways, blocking apoptosis and inhibiting matrix metalloproteinase activity [41–43]. Moreover, these drugs may alter innate immune response signaling to dsRNA in oral epithelial cells, with increased production of IL-8 and TNF and NF-κB activation [44]. It has also been shown that the choice of treatment regimen affects the levels of immunological biomarkers IP-10 and MIG, which are chemokines induced by interferon-γ. The levels of both biomarkers were reduced after treatment, however their decline was significantly smaller with ATV/r (Protease Inhibitor) than with EFV (NNTRI), while no significant difference was found between LPV/r (Protease Inhibitor) and EFV or between other first-line drugs [45]. Similarly to what we found in our cohort, patients in an LPV/r based therapy presented IFN-α and MCP-1 levels similar to the baseline [46] after six years of treatment, indicating a partial failure in decreasing immune cell activation.

Patients with slow disease progression, under HAART 2, persisted in an immune activation status that was not observed in patients who received HAART 1 (Fig 3). The complete distinct immunological biomarker network generated by HAART 2 patients (Fig 4) can be explained by the ability of LPV/r and ATV treatments to directly modulate cytokine response and transcription factor expression, as seen by others [47].

IP-10, TNF-α, IFN-α, IL-6, and IL-10 were the best immune biomarkers to identify patients according to treatment (Fig 5A). Based on this finding, it is safe to say that while first-line therapy (HAART 1) is able to recover immune function to a profile very similar to that observed in non-infected individuals, patients under HAART 2 presented an inflammatory biosignature profile very close to that of untreated patients.

In fact, we found that IP-10>TNF-α>IFN-α were crucial biomarkers to segregate the groups (Fig 5B). These biomarkers have an important role in HIV infection, as follows: High IP-10 levels in HIV are critically related to immunological activation [48] and recognized as strongly predictive of rapid HIV disease progression [10]. Type I interferons are well known for their anti-viral functions, which are considered as essential in controlling viral infections. However, during persistent viral infections, such as that occurring in HIV infection, prolonged IFN-α activity directly contributes to increased immune activation and dysfunctional activity of monocytes, T-cells, and B-lymphocytes. Moreover, there is an association of increased IFN-α activity and the development of non-AIDS-related events [49]. Because monocyte activation is a key event in systemic inflammation during HIV infection, cytokines produced by these cells such as IL-6 and TNF-α as well as sTNFR-1, the soluble receptor 1 for TNF, are strongly associated with disease progression in the HAART era [50–53].

Antiretroviral therapy spreading brings new perspectives to HIV-infected patients. Our study and previous work done by others [54] reinforce the importance of establishing new immunological biomarkers beyond the virological ones, but further may reveal drawbacks associated to drug therapies, such as the fact that these drugs may modulate immune response. Promising biomarkers such as the ones shown here are relatively easy to standardize and could give rise to a novel perspective into disease outcome, opening new avenues into novel therapies that consider patients under different HAART regimens as a heterogeneous group of individuals in which distinct outcomes may be expected.

## Supporting Information

S1 Fig Cut-off thresholds used to segregate subjects according to low or high levels of serum biomarkers. (TIFF)

S2 Fig Cut-off thresholds of biomarkers used to segregate HIV+ patients into those with putative slow and rapid disease progression. (TIFF)

S3 Fig Agreement of biomarkers used to segregate HIV+ patients into those with putative slow and rapid disease progression. (TIFF)

S4 Fig CD4+ T cell levels before (Nadir) and after treatment of HIV+ patients under different antiretroviral regimens. (TIFF)

S1 Table Regimen details of treated patients. (PDF)

S2 Table Basic characteristics of HIV-1-infected individuals and healthy donors. (PDF)

S3 Table Detailed information about patients in each HAART treated group, including duration of infection, number of years under treatment, and nadir CD4+ T cell counts. (PDF)

S4 Table Spearman correlation test of CD4+ T cell counts, nadir CD4+ T cell counts, duration of infection, and duration of treatment versus immunological plasma biomarkers in patients under different antiretroviral treatments. (PDF)
