# Supplementary figures and images for: Dysregulated Immune Activation in Second-Line HAART HIV+ Patients Is Similar to That of Untreated Patients
Source: PLoS One. 2015 Dec 18;10(12):e0145261. doi: 10.1371/journal.pone.0145261 (PMC4684276; doi:10.1371/journal.pone.0145261)

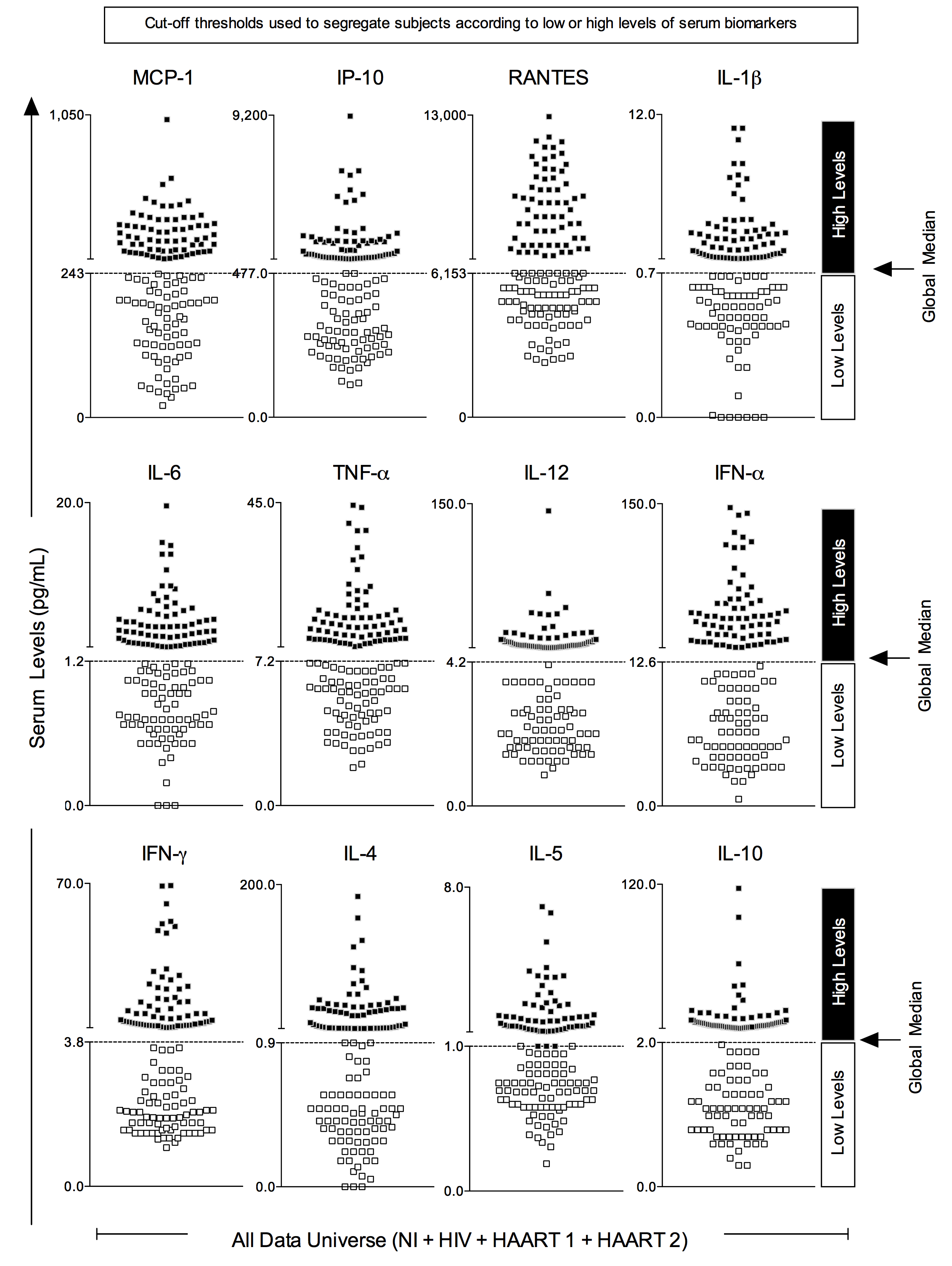

Supplement: S1 Fig — (TIFF) [file pone.0145261.s001.tiff]

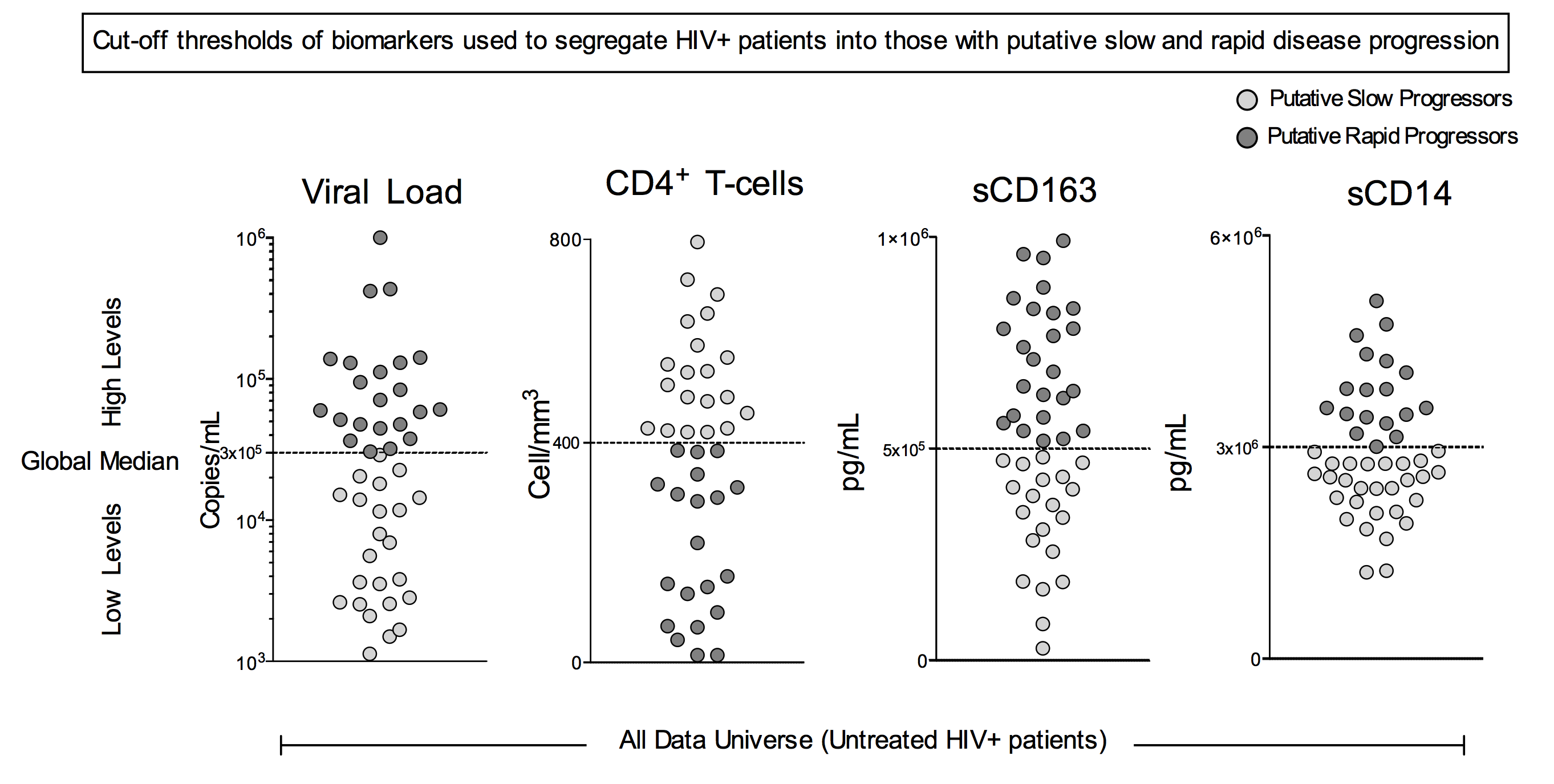

Supplement: S2 Fig — (TIFF) [file pone.0145261.s002.tiff]

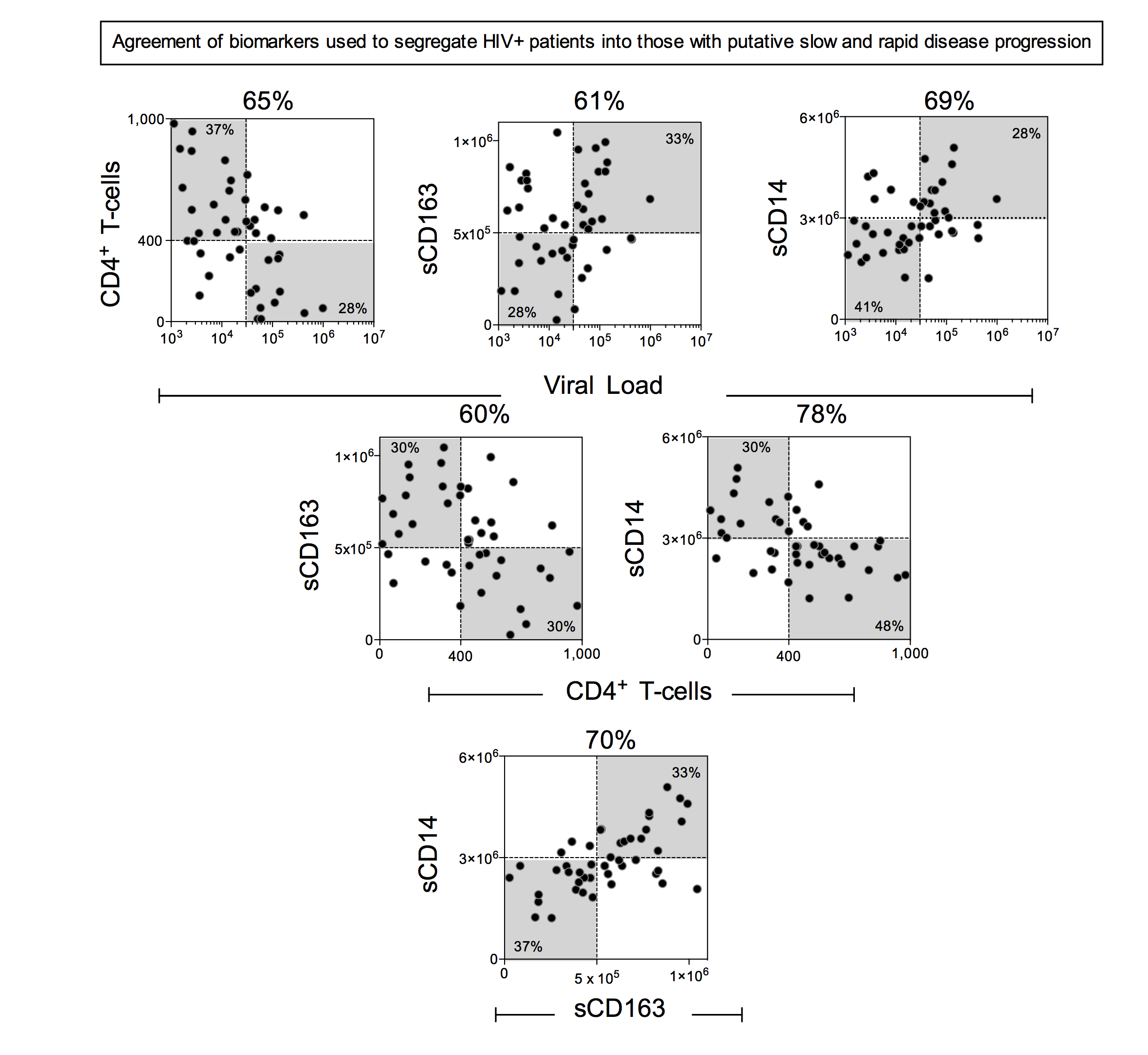

Supplement: S3 Fig — (TIFF) [file pone.0145261.s003.tiff]

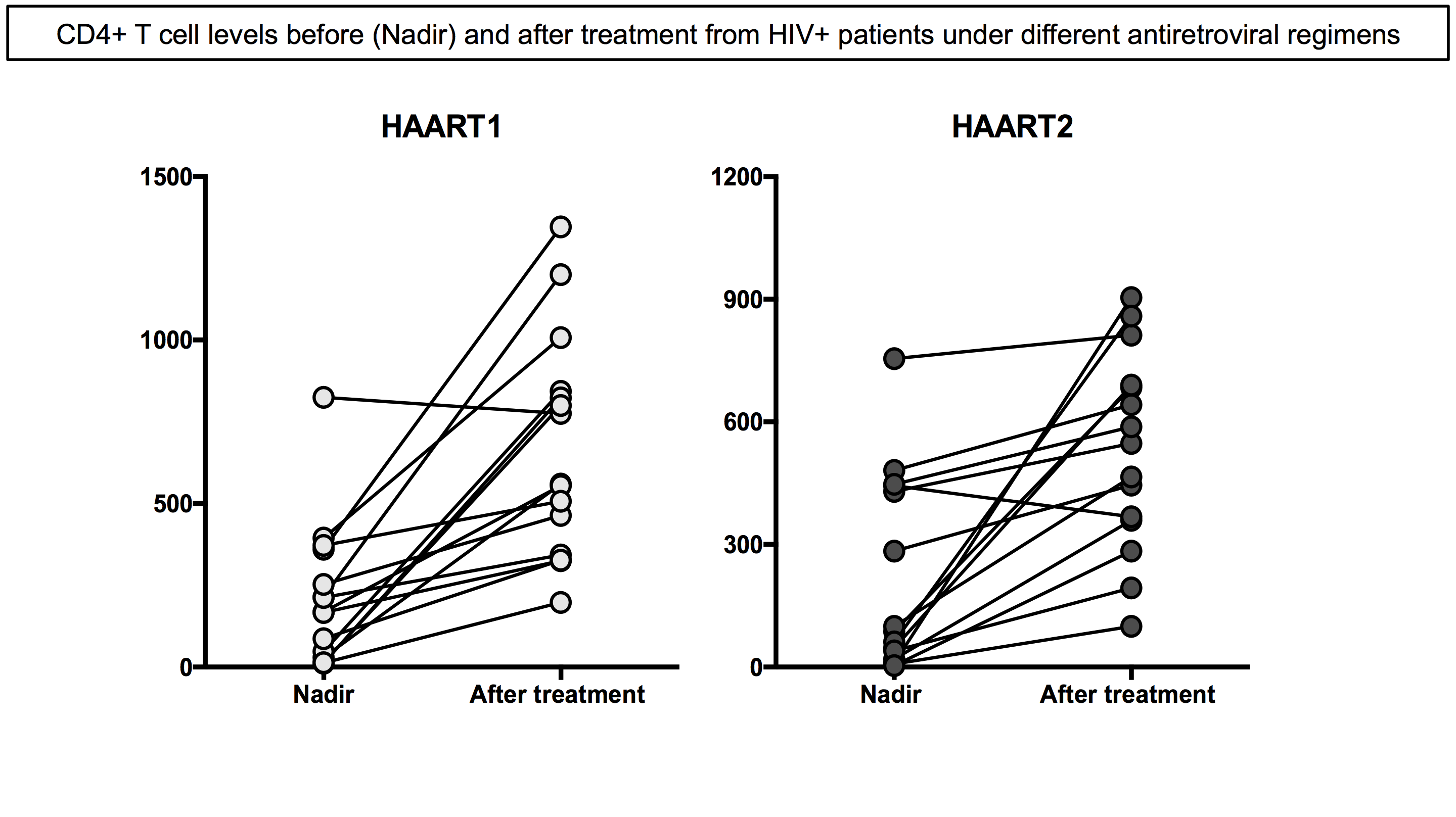

Supplement: S4 Fig — (TIFF) [file pone.0145261.s004.tiff]
