# Supplementary material for: Dysregulated Immune Activation in Second-Line HAART HIV+ Patients Is Similar to That of Untreated Patients
Source: PLoS One. 2015 Dec 18;10(12):e0145261. doi: 10.1371/journal.pone.0145261 (PMC4684276; doi:10.1371/journal.pone.0145261)
Supplement: S1 Table — (PDF) [file pone.0145261.s005.pdf]

S1 Table. Regimen details of treated patients.

Drug Combination	*(n)*
**First-line regimen (HAART 1)**	
AZT + 3TC + EFV	10
TDF + 3TC + EFV	5
**Second-line regimen (HAART 2)**	
TDF + 3TC + LPV/r	5
TDF + 3TC + ATV/r	4
AZT + 3TC + LPV/r	3
AZT + 3TC + IDV	1
AZT + ATV/r	1
TDF + 3TC + FOS-APV	1

AZT: zidovudine; 3TC: lamivudine; TDF: tenofovir; EFV: efavirenz; LPV/r: lopinavir + ritonavir; ATV/r: atazanavir + ritonavir; IDV: indinavir; FOS-APV: fosamprenavir; HAART: highly active antiretroviral therapy
