# Supplementary material for: Dysregulated Immune Activation in Second-Line HAART HIV+ Patients Is Similar to That of Untreated Patients
Source: PLoS One. 2015 Dec 18;10(12):e0145261. doi: 10.1371/journal.pone.0145261 (PMC4684276; doi:10.1371/journal.pone.0145261)
Supplement: S2 Table — (PDF) [file pone.0145261.s006.pdf]

S2 Table. Basic characteristics of HIV-1-infected individuals and healthy donors.

Baseline characteristic	Value			
	Healthy controls (NI)	HIV		
		Untreated	HAART1	HAART2
Subjects (n)	66	46	15	15
Female [no. (%)]	33(50)	14 (30)	4 (27)	4 (27)
Male [no. (%)]	33 (50)	32 (70)	11 (73)	11 (73)
Age [yr (range)]	35,2 (25-45)	37 (31-44)	42 (32-47)	44 (37-54)
CD4/mL (range)	NA	465 (248-611)	672 (343-843)	529 (359-690)
HIV RNA/mL [log (range)]	NA	4,041 (3,41-4,76)	<50	<50
