# Supplementary material for: Dysregulated Immune Activation in Second-Line HAART HIV+ Patients Is Similar to That of Untreated Patients
Source: PLoS One. 2015 Dec 18;10(12):e0145261. doi: 10.1371/journal.pone.0145261 (PMC4684276; doi:10.1371/journal.pone.0145261)
Supplement: S3 Table — (PDF) [file pone.0145261.s007.pdf]

S3 Table. Detailed information about patients in each HAART treated group, including duration of infection, number of years under treatment, and nadir CD4+ T cell counts.

Patient	Duration of infection (years)	Years under treatment	CD4 at the moment of treatment
**HAART1**			
1	10	7	30
2	17	3	169
3	20	8	13
4	17	7	167
5	17	17	825
6	12	5	395
7	12	11	215
8	12	7	48
9	15	14	12
10	5	3	361
11	9	N.A.	213
12	9	9	14
13	8	8	87
14	4	4	253
15	8	8	372
Mean (interquatile range)	11.67 (8-17)	7.93 (4.75-9.5)	211.60 (30-361)
**HAART2**			
1	5	5	87
2	4	4	20
3	12	5	8
4	18	3	284
5	4	3	481
6	10	10	47
7	17	17	100
8	6	5	10
9	4	4	444
10	19	19	755
11	17	19	430
12	9	9	447
13	8	8	62
14	15	15	40
15	4	4	4
Mean (interquatile range)	10.13 (4-17)	8.67 (4-15.5)	214.6 (20-444)

N.A.- Information not available
