# Supplementary material for: Dysregulated Immune Activation in Second-Line HAART HIV+ Patients Is Similar to That of Untreated Patients
Source: PLoS One. 2015 Dec 18;10(12):e0145261. doi: 10.1371/journal.pone.0145261 (PMC4684276; doi:10.1371/journal.pone.0145261)
Supplement: S4 Table — (PDF) [file pone.0145261.s008.pdf]

S4 Table. Spearman correlation test of CD4+ T cell counts, nadir CD4+ T cell counts, duration of infection, and duration of treatment versus immunological plasma biomarkers in patients under different antiretroviral treatments.

CD4+ T cell counts versus												
	IFN-α2	IFN-γ	IL-10	IL-12	IL-1β	IL-4	IL-5	IL-6	IP-10	MCP-1	RANTES	TNF-α
**HAART1**												
Spearman r	-0.1986	-0.025	-0.4204	-0.2093	-0.2165	-0.1395	-0.5058	-0.0376	-0.1698	-0.4536	0.1058	0.1004
P (two-tailed)	0.475	0.9308	0.1194	0.4509	0.4353	0.6176	0.0565	0.8952	0.5426	0.0915	0.706	0.7204
**HAART2**												
Spearman r	0.1576	0.2556	0.3825	0.3238	0.1843	0.1395	-0.2855	-0.084	-0.05	0.075	0.5717	0.2324
P (two-tailed)	0.5722	0.3552	0.1592	0.2374	0.508	0.6177	0.3001	0.7654	0.8626	0.7925	**0.0282**	0.4015

Nadir CD4+ T cell counts versus												
	IFN-α2	IFN-γ	IL-10	IL-12	IL-1β	IL-4	IL-5	IL-6	IP-10	MCP-1	RANTES	TNF-α
**HAART1**												
Spearman r	0.3256	0.3914	0.1574	0.3739	0.4347	0.2809	0.2252	0.195	-0.0018	-0.3214	0.3247	0.2619
P (two-tailed)	0.2348	0.1492	0.5727	0.1694	0.1064	0.3081	0.4166	0.4831	0.9976	0.2425	0.2362	0.343
**HAART2**												
Spearman r	-0.1987	-0.0822	0.1072	-0.2791	-0.356	-0.1717	-0.3573	-0.3539	0.0321	-0.4429	0.2957	-0.2109
P (two-tailed)	0.4746	0.77	0.702	0.3113	0.192	0.5378	0.1903	0.1947	0.9132	0.1002	0.2823	0.4474

Duration of infection versus												
	IFN-α2	IFN-γ	IL-10	IL-12	IL-1β	IL-4	IL-5	IL-6	IP-10	MCP-1	RANTES	TNF-α
**HAART1**												
Spearman r	0.2401	0.2795	0.343	0.2987	0.3818	0.4522	0.2173	0.2978	0.0487	0.146	-0.2534	-0.1629
P (two-tailed)	0.3849	0.3098	0.209	0.2765	0.1596	0.0916	0.4324	0.278	0.8627	0.6013	0.3584	0.5583
**HAART2**												
Spearman r	0.2034	0.3791	-0.0036	0.2665	0.0714	0.1915	-0.0535	0.1101	0.0956	-0.1028	0.1638	0.3457
P (two-tailed)	0.4623	0.1625	0.9909	0.3329	0.7986	0.4895	0.8487	0.6928	0.7332	0.7137	0.5552	0.2049

Duration of treatment versus												
	IFN-α2	IFN-γ	IL-10	IL-12	IL-1β	IL-4	IL-5	IL-6	IP-10	MCP-1	RANTES	TNF-α
**HAART1**												
Spearman r	0.1306	-0.1594	-0.2684	-0.0281	-0.0372	-0.049	-0.2437	-0.3074	0.0661	0.496	0.0027	-0.3046
P (two-tailed)	0.6398	0.5667	0.3302	0.921	0.8952	0.8618	0.3779	0.2626	0.8137	0.062	0.9934	0.2673
**HAART2**												
Spearman r	-0.0108	0.0622	-0.3192	0.0984	-0.1444	-0.13	-0.4819	-0.2561	-0.2415	0.236	0.5561	0.1208
P (two-tailed)	0.9702	0.8242	0.2438	0.7247	0.604	0.641	0.0704	0.3532	0.3829	0.3939	**0.0335**	0.665
